# An ensemble-based enhanced short and medium term load forecasting using optimized missing value imputation

**DOI:** 10.1038/s41598-025-06610-9

**Published:** 2025-07-01

**Authors:** Tania Gupta, Richa Bhatia, Sachin Sharma

**Affiliations:** 1 Department of Electronics and Communication, Netaji Subhas University of Technology, East-Campus (formerly AIACTR, affiliated to GGSIPU, Dwarka), New Delhi, India; 2 Netaji Subhas University of Technology, East-Campus, New Delhi, India; 3 Department of Electrical and Electronics Engineering, Manipal Institute of Technology, Manipal Academy of Higher Education, Manipal, Karnataka India

**Keywords:** Advanced metering infrastructure, Ensemble method, Load forecasting, Load profiles, Missing value imputation, Machine learning, Smart meter, Energy science and technology, Engineering

## Abstract

Electricity load forecasting is integral to planning, energy management, and the energy market. Utility companies serve a massive number of customers by supplying electricity. These utility companies require a precise forecast of electricity usage. This paper presents a forecasting model for energy load based on the ensemble voting regressor method. In addition, to enhance the accuracy of forecasting, develop an imputation method for handling missing values in the user’s energy consumption data. A real-time data set is used for performance comparison with multiple imputation techniques to validate the imputation approach by generating random missing data for different missing rates of 10–30%. The proposed forecasting model is compared with other state-of-the-art methods to show its effectiveness in terms of MAPE, MAE, and RMSE. The experimental results demonstrate that the proposed methodology significantly improves the accuracy of the predicted load for a day and week ahead of energy consumption.

## Introduction

**Fig. 1:**
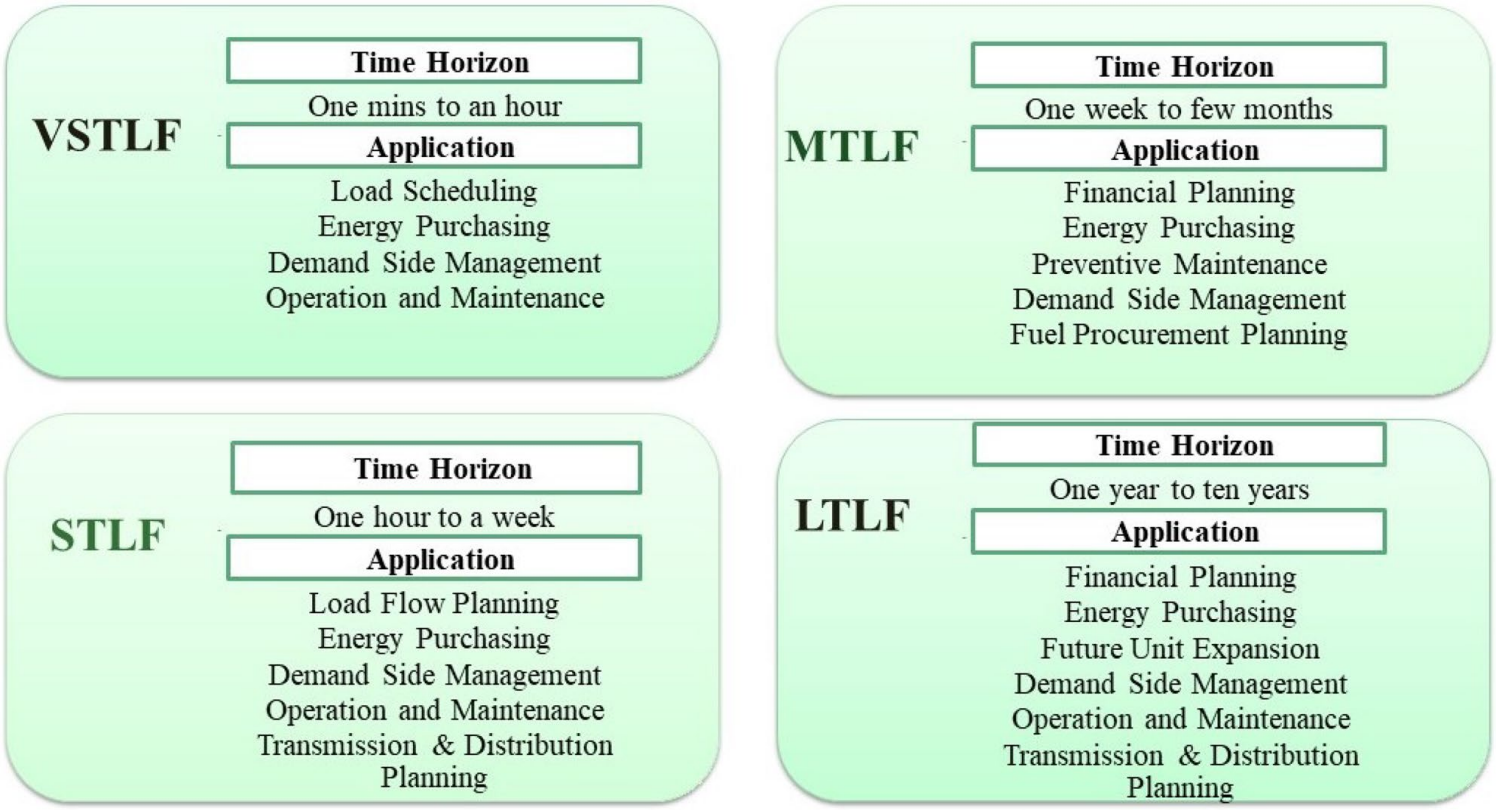
Different forms of Load Forecasting based upon time horizon (VSTLF, STLF, MTLF and LTLF) and their applications..

Between 2015 and 2040, worldwide energy consumption will rise by 28%^1^. The need for energy power is growing rapidly, yet the available resources are running out at an alarming pace. To maximize its use and reduce production costs and environmental risks, energy sources should be managed effectively^2^. Load planning is essential to energy management and grid operation as it helps to use resources more efficiently, keeps the grid stable, and helps people make decisions about their consumption^3^. Smart grids employ load forecasting (LF) by analyzing user power consumption data collected through smart energy meters^4^. Smart meters are an important part of smart grid technology which can record how much electricity a customer uses at regular times by connecting to communication networks. The advanced metering infrastructure (AMI) enabled us to monitor and analyze much new information about the energy usage of consumers^5,6^. Based on the temporal horizon, there are four forms of load forecasting in smart grids. Figure1 contains a detailed description of each form with its time frame and applications^7^.

## Motivation

Accurate power demand estimates at more minor time scales are important for optimizing generation, planning repair, and ensuring a steady energy flow. In this context, a number of studies have been attempted to predict short-term load by using machine learning models. With the widespread use of smart meters, there is now a lot of high-resolution data on how much energy is used, which could help improve the accuracy of intraday/weekly load forecasts. However, missing data presents a substantial obstacle when using smart meter data for load forecasting. Missing data can result from several things, including poor communication, broken equipment, or mistakes in data capture^8^. Data gaps can inject bias and mistakes into forecasting models, lowering the efficacy of those models^9^. As a result, it is critical to give attention to techniques that can manage missing data well while enhancing the precision of load forecasting.

This paper offers a solution to this issue by enhancing load forecasting accuracy of electric load using a combination of ensemble voting regressor prediction models with properly counter the missing values with an efficient imputation technique. Three distinct machine learning (ML) prediction algorithms integrate with an ensembling model for a more reliable and precise prediction. A hybrid ensemble technique improves forecasting performance by utilizing the variety and complementary capabilities of these algorithms along with an efficient technique for addressing missing data. This paper conducts a comparative study of different missing value imputation techniques; chooses the best one; and applies it to different prediction models to estimate the energy load. To fill in the missing value, this paper proposes spline interpolation. By using this method, missing data will less negatively impact the forecasting process, and the load estimates will be more accurate.

In this research, three regression models, random forest (RF), extra tree (ET), and decision tree (DT), ML algorithms are ensembled which are considered to be a better method than using a single ML algorithm. The outputs of these regression models are combined using simple averaging to provide a single forecast, referred to as the ensemble voting regressor (EVR). This approach seeks to improve load forecasts’ accuracy, robustness, and reliability by combining multiple models and effectively handling missing data, facilitating more informed decisions for energy management and grid operation.

The remainder of the paper is organized as follows: The Section “State of art methods and our contribution” provides an overview of existing literature related to this study and the contribution of this paper. Section “Network model” discusses this study’s network model used for LF. Section “Proposed methodology” discusses the different stages of the proposed model. The Section “Evaluation metrics” describes the evaluation metrics and the implementation of the proposed method. The results and analysis of the experiments conducted on the real-world smart meter dataset are presented in Section “Simulation results and discussions”. Section “Conclusion” concludes the paper with a summary of the findings and future research directions of this work.

## State of art methods and our contribution

Electric utilities rely heavily on short-term to medium-term LF, allowing them to make educated decisions about power procurement, generation, load switching, and infrastructure development. Several methods have been developed in the literature on residential short-term load forecasting problems for using hierarchical data^7^. In real-world time series datasets, missing values are a common problem due to various factors like sensor malfunction, communication problems, and other technical reasons. In particular, electricity consumption datasets collected with smart meters may have missing data resulting from hardware failures, network disruptions, or maintenance activities, leading to incomplete load profiles that obstruct energy management and planning. This poses serious problems as it can disrupt the temporal continuity and patterns essential for accurate analysis and reduce the accuracy and reliability of forecasting algorithms^10,11^ .

Despite this, addressing missing values has received little attention in electric load forecasting. Researchers often begin with more straightforward methods and advance to more complex strategies to adequately address this issue in time series data^12,13^. The transformer network applied for imputing the missing values of energy data for three categories of missing data (MAR, MCAR, and MNAR) and compared with other data-driven methods produces better results for different % of missing values, but at the cost of increasing the system’s complexity^12^. Different statistical imputation techniques were applied to the numerical data on five different types of datasets using data mining^14^.

In^15^, the author addresses the missing value in solar PV time series datasets using the modified column mean imputation (CMI) and further forecasts the solar power using XGBoost. The author investigates the integration of spline interpolation imputation method with ARIMA model, which enhances the forecasting accuracy of stock prices and the ability to capture smooth transitions in financial time series data^16^. Statistical and data-driven methods are applied for missing data of residential buildings by considering the missing completely at random (MACR)^17^. The six-stage PSO technique is applied to the missing value imputation, the complex one^18^. The author reported that with consideration of missing value imputation, the forecasting error of EV charging stations decreased by 9.8%^9^; these results raise the need for special attention to the missing values in the data and their impact on forecasting of electric load.

Further in this section, a quick review of the literature on load forecasting and the contribution of this study is provided. Early on, these forecasts or predictions were made utilizing standard or conventional mathematical methods. These methods have been strengthened due to research for more accurate forecasting in numerous disciplines of study and the creation of improved instruments. Regression, exponential smoothing, multiple regression, and support vector regression are the conventional forecasting techniques^19^. The regression method is a statistical technique employed to predict the future values of a variable using other variables. Additionally, it helps identify the relationship between dependent variables and one or more independent variables^20^. To analyze seasonal fluctuations and fit the data, Barakat tested the regression model^21^. In^22^ the author used a sliding window-based ARIMA algorithm for forecasting the energy consumption of residential and industrial consumers. Traditional load forecasting methods frequently struggle to account for temporal dynamics and hierarchical structures, manage massive data, add external influences, and capture complicated and non-linear interactions. Accurate forecasting, flexibility to shifting conditions, and the capacity to quantify uncertainty are all hampered by these restrictions. As a result, novel strategies utilizing deep learning models, machine learning algorithms, sophisticated statistical techniques, and ensemble methods have been created to address these issues. The deep learning method arises and makes a significant breakthrough as computational resources and meter data increase. The ability to capture complex patterns, temporal dependencies, and hierarchical relationships, along with improved accuracy, scalability, and flexibility, provided by these new approaches enables electric utilities to make better decisions and meet the changing demands of load forecasting in the energy sector. Table 1 provides a comprehensive summary of the related work.

**Table 1:** Existing literature related to missing value imputation and load forecasting.

Paper	Year	Algorithm/Method	Dataset	Validation	Purpose
Lotfipoor et al.^12^	2023	Clustering/Transformer network	UK with missing rate 10–30%	RMSE	Missing value imputation (electric power application)
Kim et al.^17^	2023	Data driven method	From South Korea with missing rate 10–50%	NRMSE	Missing value Imputation (residential electric power application)
Benitez et al.^15^	2023	Statistical CMI method	Solar radiance dataset with missing rate 5–20%	RMSE, MAE	Missing value impuation (solar power time-series)
Yu^16^	2023	Spline interpolation model	Stock exchange dataset	–	Missing Value Impuation (stock market time-series)
Hemanth et al.^18^	2022	PSO	Realtime data set from Indian institute	RMSE	Missing value imputation (electric power application)
Lee et al.^9^	2020	combination of spline interpolation and EM algorithm	EV charging station data from Korea Electric Power Corporation (KEPCO)	MAE	Missing value impuation (electric vehicle charging forecasting)
Haydar et al.^23^	2018	Interpolation & Kalman Filtering	Australian Bureau of Meteorology (BOM) solar radiance dataset	RMSE, MAE, MASE	Missing Value Impuation (solar power time-series)
Poulos et al.^13^	2018	Classification model	UCI repository	–	Missing value imputation (mixed dataset for different application)
Atef et al.^24^	2022	Bi-LSTM DNN	Residential house data from France	MAPE, MAE	One day ahead LF
Goehry et al.^25^	2020	Clustering	Irish smart meter dataset	RMSE, MAPE, MAE	One day ahead LF
Moon et al.^26^	2020	Stacking ensemble approach	Energy data set from Korea Electric Power Corporation (KEPCO)	MAPE, MAE	One day ahead LF
Deng et al.^27^	2019	TCMS-CNN	Ireland Energy data	RMSE, MAPE, MAE	Two day ahead LF
Nepal et al.^19^	2019	Regression and moving averages	Electricity data of Chubu University, Japan	RMSE, MAPE, MAE	One day ahead LF
Alberg et al.^22^	2018	Moving averages with sliding window	Real data from Israeli city	MAPE	One day ahead LF
Shi et al.^28^	2018	PDRNN	Ireland energy grid	RMSE, NRMSE, MAE	One day ahead LF

In^29^, Misiti et al. employs a three-stage process. They use wavelets first to preprocess the load data from each consumer. After that, they separate the customers into several groups. Finally, depending on a set of criteria, they merge to generate more significant clusters. They conclude that the best outcomes are typically obtained with a minimal number of clusters. Their case study demonstrates that utilizing 19 clusters produced the best outcomes. In^30^, Alzate and Sinn employ extracted features using wavelet analysis, Kernel Spectral Clustering for clustering, and PARX as their forecasting technique. In^25^, the developed approach consists of building experts using random forests trained on some subsets of customers, then normalizing their predictions and aggregating them with a convex expert aggregation algorithm to forecast the system load. Other statistical learning methods, such as SVM and random forests, are also applied to the STLF, and the feature engineering, which includes weekdays and temperatures, is utilized to its most significant potential in order to feed SVM and random forests^31,32^. The deep learning models are stacked together using a meta-regression model presented in^26^. The most popular deep learning methods are long short-term memory (LSTM) and convolutional neural networks (CNN), which have recently gained popularity in power load forecasting as well^24^.

In^27^, a deep convolutional neural network model based on multi-scale convolutions is presented for multi-step short-term load forecasting. CNN can efficiently extract depth characteristics and filter noise^33^. To deal with the significant volatility and unpredictability of in-home load forecasting, Shi et al.^28^ introduced a pooled deep recurrent neural network (PDRNN), which reduced the RMSE by 6.5% compared to the traditional RNN forecasting model. In^34^, the author proposes techniques for precise load forecasting in distribution networks, which are essential for maintaining grid security and effective power system administration. The integration of LSTM and GRU models is the main focus. It is validated using the IEEE-33 bus power distribution network. In^35^, the author presents the performance of several wind forecastings models, such as ARIMA, LSTM, Random Forest, and XGBoost, in comparison to one another. The results suggest that the XGBoost performs better than the others in accurately forecasting wind speed. In^36^ the author proposed a Bi-GRU prediction model to forecast short-term residential energy consumption and compared it with other models like LSTM, GRU, and Bi-LSTM. Chaodong et al. propose an ensemble deep learning model based on GRU and kernel ridge regression (KRR) for predicting short-term industrial load^37^. Experimental results on an industrial Korean data set proved that the proposed model outperforms the generalized models such as SVM, ELM, and CNN. A multi-stage ensemble model based upon GRU for LF is presented in this study with an emphasis on improvement in accuracy and optimization. The forecasting models’ performance is improved by the use of a multi-objective evolutionary method^38^.

The use of deep learning for load forecasting has its challenges, including overfitting, poor assessment of uncertainty, model selection, managing noisy data, and interpretability. To overcome these issues this paper presents an ensemble method-based load forecasting with special attention to the non-reported data. Ensemble approaches reduce overfitting, improve generalization, and produce reliable forecasts by merging numerous deep learning models trained on various subsets of data or using various architectures. In addition, they overcome problems associated with model selection by employing a hybrid approach. By averaging the prediction of three models, the effect of noise is mitigated in the ensemble approach. Additionally, the interpretability and explanation of load projections are enhanced by choosing simpler ML models for ensembling. The constraints of deep learning approaches for load forecasting are efficiently overcome by ensemble methods via these factors. The main contribution of this paper is as follows:

1. Proposed the method for missing data imputation for electricity data of absent values shows effectiveness compared to other methods.
2. The analysis with varying missing rates, ranging from 10 to 30%, demonstrates that the performance of the proposed imputation method is not significantly affected by the percentage of missing values.
3. This study adopts the ensemble method for short-term and medium-term load forecasting, which is compelling in time series forecasting.
4. The proposed prediction model integrates the features of three ML regression models in ensemble-based forecasting to form an ensemble voting regressor (EVR). K-fold cross-validation was applied to individual ML models to evaluate the performance of individual models by splitting the dataset into 10 subsets.
5. This EVR outperforms individual models by combining the estimation of three models. The proposed scheme is developed to predict energy load one day and one week ahead successfully.
6. An empirical data set of 5000 consumers grid is used and validated to show the effectiveness of the proposed scheme.
7. The proposed method is compared with other baseline models to show its effectiveness in terms of MAPE, MAE, and RMSE.

## Network model

**Fig. 2:**
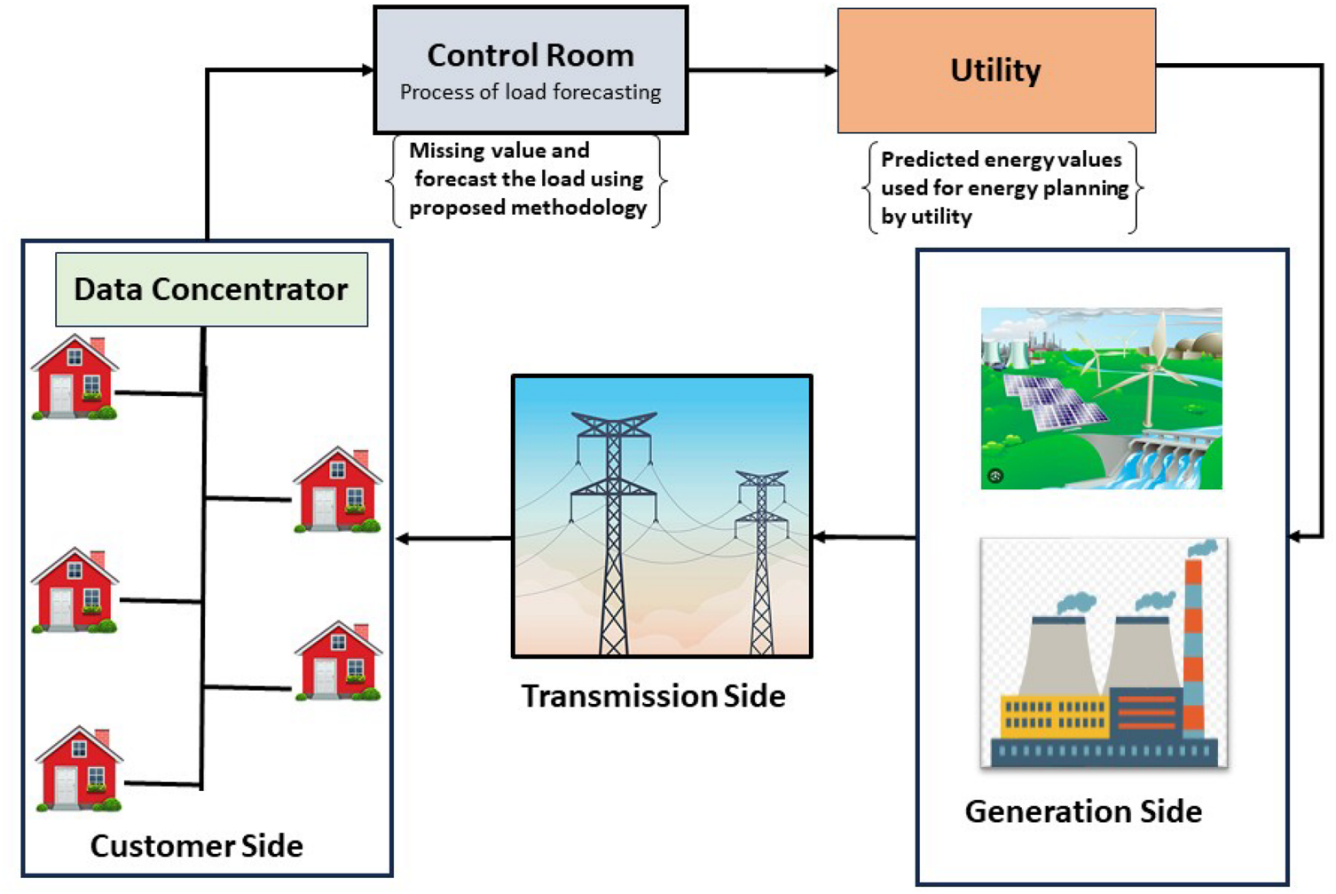
Network Model showing the process of load forecasting by collecting data from smart meters, used for forecast load and give back to power transmission and distribution unit through utility. This figure is composed by the author in Microsoft power-point using freely available public domain images. Sources include istockphoto^39,40^ and other open-access image repositories^41,42^.

The smart grid mainly has three primary elements–generation, transmission, and AMI that comprise the smart grid. The inclusion of AMI in the smart grid is crucial. The three main components of AMI are the smart meter, the data concentrator, and the control room. AMI allows two-way communication, remote smart meter management, and data collection for billing and rate setting. In addition, a smart meter records information from the customer’s home electrical appliances. The control room will get this information once it has been relayed to the data concentrator. The smart grid network architecture depicted in Fig. 2 includes all appliances in a house that communicate with a HAN smart meter. Electricity use data would be sent over NAN from the proposed smart meters with technology, and all the data processing for load prediction would be done in the control room.

The control room receives this compiled information through WAN . Data analysis would be aided by the information gathered in the control room. If some energy points are missing or non-reported for any reason, they will be filled using a missing value imputation method. The proposed forecasting algorithm will then use the processed data. The utility will receive the predicted values to aid energy planning and transmission decision-making. Further, this load prediction can be used for demand side management, energy purchasing, and operation and maintenance.

## Proposed methodology

**Fig. 3:**
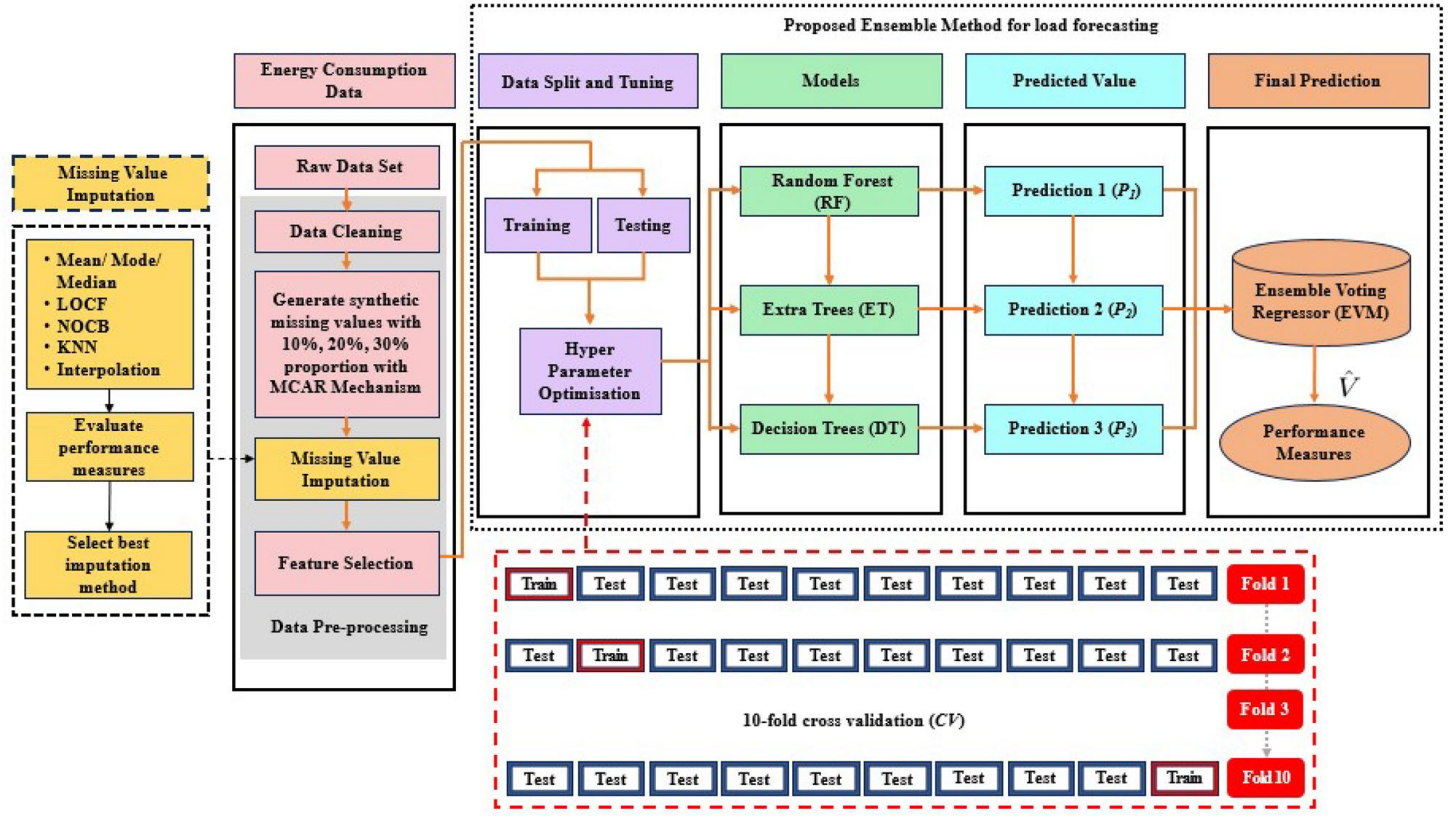
The flow of a process of the proposed model to predict load.

This paper presents a combined approach for load forecasting in consideration of the missing values present in the dataset. This approach is suitable for effectively predicting the electricity consumption for short-term (one day) and medium-term (one week) LF. The proposed methodology is divided into data preprocessing, missing value imputation, and load forecasting models; the complete flow of these subsections is given in Fig. 3.

### Data preprocessing

The smart energy data from the Irish Smart Energy Trial^43^ is used. Electric Ireland and the Sustainable Energy Authority of Ireland (SEAI) released this dataset. It includes a half-hour power report on the consumption of over 5000 Irish households and businesses. Customers who registered in the experiment had a smart meter installed in their homes and chose to participate in the study. This dataset is an amazing source of research in smart meter data analysis due to its large quantity and variety of clients, availability to the public, and lengthy duration of observations.

Let *C* be the hourly power consumption of the consumer, which is represented in matrix form as given in the Eq. (1). The cumulative electric power of every day for 24 hours has been calculated.

1 $$\begin{aligned} \begin{bmatrix} C_{1}(1,1) & C_{2}(1,1) & C_{3}(1,1) & . & . & C_{n}(1,1) \\ C_{1}(1,2) & C_{2}(1,2) & C_{3}(1,2) & . & . & C_{n}(1,2) \\ ...& ... & ... & ... & ... & ...\\ C_{1}(1,h) & C_{2}(1,h) & C_{3}(1,h) & . & . & C_{n}(1,h) \end{bmatrix} \end{aligned}$$

where $C_{1}(1,1)$ is the electricity consumption of the first consumer for the first day’s first hour. The $C_{n}(1,h)$ shows the electricity consumption of $n^{th}$ consumer for the first day *h* hour.

It is impractical to input raw data directly to a prediction model, as it is typically in an insufficient form, would result in certain errors or inaccurate predictions. Therefore, pre-processing is a crucial step before the data can be utilized effectively in a model. It transforms the raw data into a useful and effective form by modifying or removing redundant or corrupt data. After the cleaning process, the next step involves identifying and addressing missing or null values within the dataset discussed in the next part.

#### Missing value imputation

Electricity consumption datasets collected with smart meters may have missing data resulting from hardware failures, network disruptions, or maintenance activities, leading to incomplete load profiles that obstruct energy management and planning. This poses serious problems as it can disrupt the temporal continuity and patterns essential for accurate analysis and forecasting^10^; therefore, they must be filled in using an appropriate imputation procedure.

There are three categories of missing data: MAR(missing at random), MCAR(missing completely at random) and MNAR(missing not at random)^14^. This study considers the MCAR category of missing data^44,45^, which states that the probability of a missing point is independent of both observable and unobserved data. This assumption allows the application of different imputation techniques without causing significant bias. As outlined in the data preprocessing stage of Fig. 3, raw data is cleaned for all the outliers, ensuring completeness, resulting in a fully observed dataset with no missing values that serves as the baseline for this study. Artificial missing values are introduced using the MCAR assumption at distinct proportions of 10%, 20%, and 30% across the dataset to do the evaluation, which is inspired by the study^12,17,45^.

This paper proposes the cubic spline interpolation method to fill missing values by analyzing different imputation techniques. Interpolation using splines is a prevalent method for filling in absent values in a dataset. It estimates the missing values by constructing a uniform curve through the observed data points. The spline function based on data points has been calculated. When missing data is encountered, the spline function will make a reasonable assumption based on the surrounding information. The cubic splines have been interpolated to fit the spline curves to the observed data points. The spline functions are then evaluated at the positions of the missing values to obtain the interpolated values. Lastly, the missing value is replaced by the interpolated values. Mathematically, let’s say for a given function $C=f(h)$ from known data points $(h_0,C_0),(h_1,C_1),(h_2,C_2),.....,(h_n,C_n)$. Specific data point $(h_i, C_i)$ is referred to as a knot through which the interpolated curve must pass. These knots divide the domain into intervals $[h_i,h_{i+1}]$, over which individual cubic polynomials are defined^16^.

Let’s consider the segment between the coordinates $(h_i, C_i)$ and $(h_{i+1}, C_{i+1})$. For this segment, we define a cubic polynomial function as:

2 $$\begin{aligned} S_i{(h)} = a_{i} + b_{i}(h - h_{i}) + c_{i}(h - h_{i})^2 + d_{i}(h - h_{i})^3 \quad \text {for } i=0,1,...,n-1 \end{aligned}$$

subject to the conditions:

- *Condition 1:* spline must pass through the starting point of each interval: $S_i(h_i) = C_i$.
- *Condition 2:* spline must pass through the ending point of each interval: $S_i(h_{i+1}) = C_{i+1}$.
- *Condition 3:* 1st derivative continuity at interior points: $S_i'(h_{i+1}) = S_{i+1}'(h_{i+1})$.
- *Condition 4:* 2nd derivative continuity at interior points: $S_i''(h_{i+1}) = S_{i+1}''(h_{i+1})$.

Conditions 1 and 2 ensure that the spline interpolates data points accurately. Conditions 3 & 4 are continuity equations for the smoothness of the spline. $a_i, b_i, c_i \text { and } d_i$ are coefficients determined to ensure the smoothness and continuity of the spline, which is given in 3, 4, and 5.

3 $$\begin{aligned} & a_{i}= \frac{b_{i+1}-b_{i}}{3h_{i}} \end{aligned}$$

4 $$\begin{aligned} & c_{i}= \frac{k_{i}}{h_{i}}-\frac{h_{i}}{3}(b_{i+1}+2b_{i}) \end{aligned}$$

5 $$\begin{aligned} & d_{i}= y_{i} \end{aligned}$$

The cubic spline interpolation provides a precise and smooth way to estimate values between known data points by meeting these conditions^23^. This is carried out using the pandas *interpolate* function with parameter value *method= ‘spline’*, *‘order=3’*, which ensures smooth curve fitting while preserving consumption trends. The process for finding the best imputation method used in this study is shown in algorithm 1. After that, as a part of the feature engineering process, the timestamps associated with each half-hourly consumption are used to derive different time series features, including dayhour, day of week, quarter, month, year, and day of year, reflecting daily and weekly load patterns as mentioned in algorithm 1.

**Algorithm 1:**
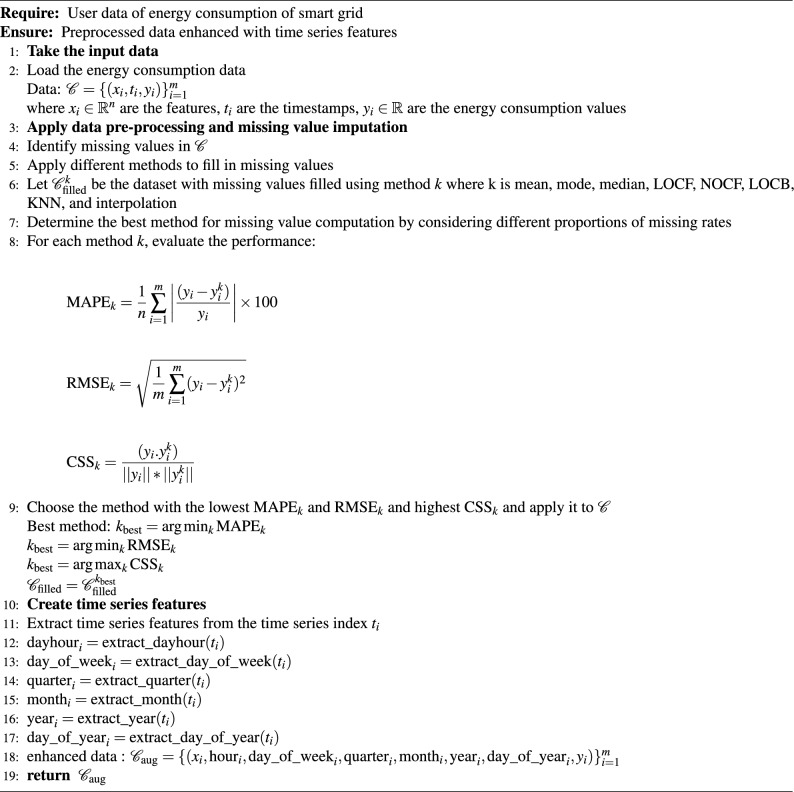
Energy consumption prediction: Data pre-processing and missing value imputation.

### Proposed ensemble method for load forecasting

This study developed an ensemble voting regression (EVR) prediction model that combines three basic ML algorithms Random Forest (RF), an extra tree (ET), and a decision tree (DT) for smart grid energy load prediction. Combining these learning methods in ensemble forecasting combines the flexibility of decision trees in capturing complex patterns with the ability of random forests to reduce overfitting and enhance generalization. The process followed for building the forecasting model is given in the following steps:

**Table 2:** Parameters chosen for configuring the individual models which are part of ensemble voting regressor.

Random forest		Extra tree		Decision tree	
Parameters	Value	Parameters	Value	Parameters	Value
n_estimators	100	Splitter	Random	Splitter	Best
min_samples_split	2	max_depth	None	max_depth	None
max_depth	None	min_samples_split	2	min_samples_split	2
max_features	Auto	min_samples_leaf	1	min_samples_leaf	1

1. The extracted features in the training data were fed to the different base machine learning models RF, ET, and DT. For configuring the individual models the selected parameters are listed in the Table 2.
2. After the computation of performance measures based on default parameter values from the training and test data, the optimal hyperparameter combinations are discovered using the K-fold cross-validation (CV)^46,47^, a popular method for this purpose. Its pictorial representation is shown in Fig. 3. The ten-fold CV technique is applied in this work, where the training set is divided into ten equal subsamples, nine of which are used as the training subset and one as the validation set. The process is repeated ten times until every subsample is utilized as a validation set.
3. Each base model training data set divided into 10 parts from $C_1$ to $C_{10}$, nine is for training and one part is for testing; repeat the iteration 10 times for each model. The mean value of the predicted output $\hat{\textrm{y}}_{j}^{l}$ given in Eq. (6) from the 10 iterations considered as the evaluation result of the model.
  6 $$\begin{aligned} \hat{\textrm{y}}_{j}^{l}=\frac{1}{k}\sum _{i=1}^{k}\textrm{P}_{ij}^{(l)} \end{aligned}$$
  The best hyperparameter values are found by calculating the average of the ten validation sets.
4. The three regression algorithms are combined to construct an ensemble voting regressor (EVR) using simple averages. The trained EVR was fitted. This EVR is used to combine or *ensembles* the estimation of three models and overperform individual models. $P_1$, $P_2$, and $P_3$ are the prediction of individual models and $\hat{V}$ is the prediction of the EVR as shown in Fig. 3. Finally, the proposed model’s performance in predicting energy load is measured in terms of MAPE, MAE, and RMSE and contrasted with the results of the independent ML algorithms. The complete algorithm showing the whole process followed for the load prediction model is depicted in algorithm 2.

**Algorithm 2:**
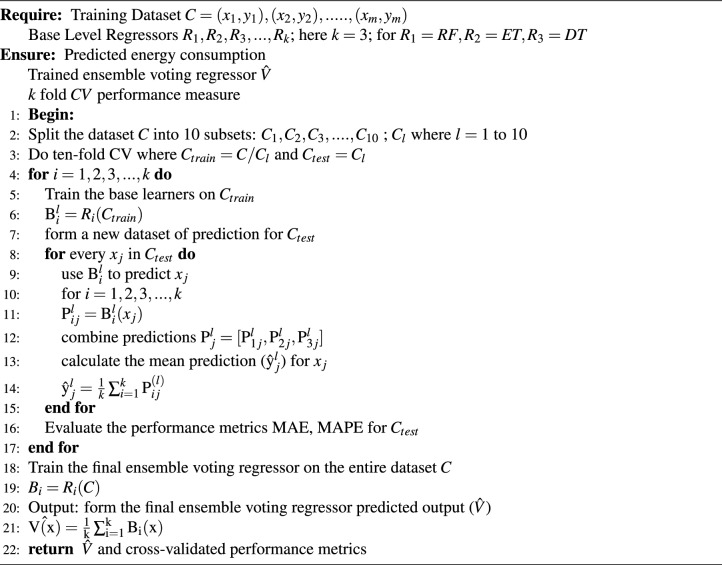
Smart grid load prediction: AI model training and ensemble voting regression (EVR).

## Evaluation metrics

This paper proposes a model for effective load forecasting. The proposed hybrid model uses spline interpolation for missing value imputation and an ensemble voting regression for load forecasting. To evaluate the performance, the model is implemented on a real-time dataset. The results are compared using different evaluation parameters.

### Evaluation metrics for missing data imputation

In order to assess the performances of missing data imputation techniques CSS, MSE , and MAPE metrics have been used. The predicted value of missing data points is compared with the actual values and based on this comparison, CSS is calculated. It extends from -1 to 1 and measures the cosine of the angle formed by the vectors. Vectors with a value of 1 are the same; with 0 are orthogonal (have no similarity), and with -1 are exactly opposite. The formula for calculating the CSS is expressed in Eq. (7) where $y_i$ is the original value and $\hat{y}_i$ is the imputed value.

7 $$\begin{aligned} CSS=\frac{(y_i.\hat{y}_i)}{||y_i||*||\hat{y}_i||} \end{aligned}$$

MSE gives a quantifiable measure of the total prediction or imputation error, making it possible to compare different models and procedures and choose the one that has the best results. Equation (8) provides a mathematical representation of MSE, the average squared difference between the actual and estimated values at time *t*.

8 $$\begin{aligned} MSE=\frac{1}{n}\sum _{i=1}^{n}(y_{i}-\hat{y}_{i})^{2} \end{aligned}$$

where *n* number of times summation iteration happens. A MAPE is a common statistic for evaluating the precision of forecasting models and imputation techniques. It calculates the average percentage deviation between imputed and actual values; demonstrating the average distance between these two values. MAPE is mathematically defined in Eq. (9).

9 $$\begin{aligned} MAPE=\frac{1}{n}\sum _{i=1}^{n}\left| \frac{(y_{i}-\hat{y}_{i})}{y_{i}}\right| \times 100 \end{aligned}$$

### Evaluation parameters for load forecasting

Several evaluation parameters can be utilized to assess the accuracy and efficiency of load forecasting models when evaluating their performance. This paper’s load forecasting evaluation parameters are MAPE, MAE, R-square, and RMSE. The dataset’s MAE and R-square at each hour are defined as given in Eqs. (10) and (11), respectively.

10 $$\begin{aligned} MAE=\frac{1}{n}*\sum \left| \hat{y}_{i}-y_{i} \right| \end{aligned}$$

11 $$\begin{aligned} R-square=\frac{n\sum y_{i} \hat{y}_{i}-\sum \hat{y}_{i}\sum y_{i}}{\sqrt{n\sum y_{i}^2-\sum y_{i}^2}* \sqrt{n\sum \hat{y}_{i}^2-(\sum \hat{y}_{i})^2}} \end{aligned}$$

## Simulation results and discussions

For the performance evaluation of the proposed method, simulations are conducted with Python 3.8.6 and Google Colab on a computer with the following specifications: Intel(R) Core(TM) i5-10210U CPU @ 1.60GHz 2.11 GHz processor, 8.0 GB RAM, 64-bit operating system, x64-based processor. In simulations, firstly, the performance is compared for the different missing data imputation techniques; on the comparison basis, select the best method, perform the load forecasting with the proposed EVR model, and compare with other short-term load forecasting models ARIMA, LSTM, and XG-Boost. The comparison table is also made to show the impact of the imputation on the forecasting.

### Performance comparison for missing data imputation technique

This section compares the efficacy of the spline missing data imputation technique used in the EVR model proposed for intraday and week-ahead load forecasting. The initial raw dataset used in this study^43^ is carefully examined for anomalies and cleansed to ensure that there are no outliers or missing values. The total of 5000 customers with half-hour consumption readings is from 2009 to 2010. This cleaned data is the original data for this study and has been further developed into three test datasets with different proportions of missing values for the imputation experiment. The selection of these particular proportions is chosen by practices in missing data research, where 10% is considered a small, 20% medium, and 30% a large amount of missing data, reflecting real-life scenarios^14,44,45^. Upon completing three test dataset preparations, six statistical imputation techniques, mean, mode, median, last observation carried forward (LOCF), next observation carried backward (NOCB), and spline interpolation, and one ML technique (KNN) are implemented to substitute the missing values. The inclusion of KNN along with the statistical method helps to benchmark the spline’s performance against a model-driven method in high-dimensional settings.

The selection of an appropriate missing data imputation technique is highly reliant on the specific characteristics of the dataset, including the nature of the variables, the mechanism of missingness, and the proportion of missing data^10,17,44^. The rationale for choosing these specific methods for comparison with the proposed method, with the strengths and weaknesses of each method in the context of time series data, is given in the Table 3. To assess the consistency in the performance of each imputation method, consider different proportions of missing rates from 1 to 30%. MAPE, RMSE, and CSS are the evaluation metrics used to determine imputation techniques’ accuracy and performance.

**Table 3:** Comparison of imputation methods with proposed method in terms of their description, strengths, weaknesses used in this study.

Imputation method	Description	Strengths	Weaknesses	Justification for selection
Mean/Median/Mode	Mean: filled by average of the data values, Mode: filled by the most repeated data value in the data, Median: filled with median or middle of data values.	Simple to apply and low computational cost	Lacks the time dependency	Frequently used due to fast and simple approach, serve as basic baseline method.
LOCF/NOCB	LOCF: Missing value is replaced by last observed value. NOCB: missing value is replaced by the next or future value.	Simple and easy to implement on a big dataset	This may distort trends during long data gaps	Used in time series data where recent data values are assumed informative
K-Nearest Neighbour (KNN)	Non-parametric approach that imputes by using values of k nearest neighbours.	Able to capture complex and non-linear patterns	Computationally expensive for large datasets.	Data-driven method to capture non-linearity in high-dimensional space.
Cubic Spline Interpolation	Filled by fitting a series of smooth, piecewise third-degree (cubic) polynomials between known data points	No need for training, extra features, handles nonlinearity and seasonal patterns	Requires sufficient values to perform	Smooth, continuous interpolation well suited to time series data.

**Table 4:** Performance comparison of imputation technique for the ISSDA electricity dataset where 10%, 20%, and 30% represents the percentage of imputed missing consumption values and shows that performance of data imputation method is independent of % of missing values in the dataset.

	10%			20%			30%		
Techniques	MAPE	RMSE	CSS	MAPE	RMSE	CSS	MAPE	RMSE	CSS
MODE	0.050	3.5802	0.955	0.087	4.6677	0.890	0.014	6.1772	0.825
MEAN	0.038	2.620	0.959	0.063	3.992	0.929	0.034	5.2262	0.848
MEDIAN	0.069	3.639	0.943	0.091	4.7224	0.882	0.019	6.8982	0.812
LOCF	0.023	1.2808	0.965	0.047	1.9189	0.943	0.076	2.3422	0.908
NOCB	0.020	1.2198	0.960	0.045	1.8750	0.943	0.068	2.3208	0.896
KNN	0.062	2.4242	0.961	0.056	3.4625	0.902	0.016	4.2267	0.845
Proposed	0.017	0.92825	0.994	0.035	1.3958	0.960	0.054	1.6587	0.937

Table 4 compares all well-known imputation methods for various performance metrics by varying missing rates in the dataset. It is clear from the results that changing the rate of missed values shows that the % of missing values in the dataset is independent of the performance of the data imputation method. The proposed method consistently obtains the highest CSS value across all scenarios, demonstrating its statistical significance advantage over other imputation methods. It consistently obtains the lowest RMSE value, demonstrating its capacity to minimize the squared differences between the imputed and actual values. The lowest MAPE value indicates its capacity to produce more precise imputations with reduced error rates.

**Fig. 4:**
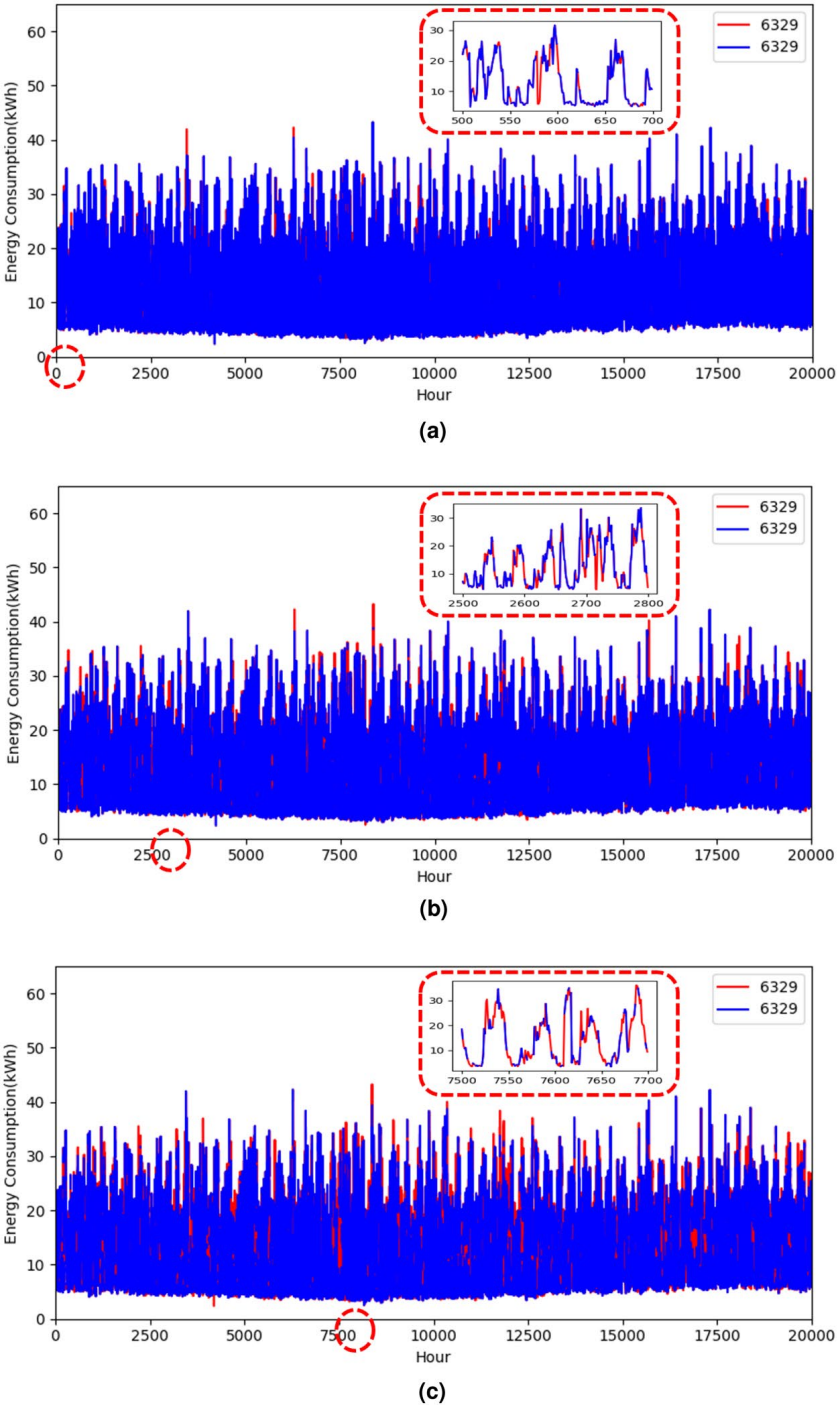
Missing data imputation graphs by taking an example for a user for different percentages of missing data values, where 6329 is the customer ID in the IRISH data set^43^.

Figure 4 illustrates an example of the imputation of missing data in the customer profile. For this purpose, a single user is identified, and a different percentage of missing values has been created and filled using the proposed method. It shows that for customer 6329 how well the missing data is imputed using the proposed method. The zoomed version of the specific segment in subplots of the Fig. 4 is shown to enhance the visual clarity of the graph. These results suggest that the proposed missing data imputation technique can significantly improve load forecasting accuracy. This enhancement in accuracy can lead to more reliable load predictions, which can help in better resource planning and management. It is an optimal method compared to the model-based approaches^17,18,48^ due to its low computation cost, fast execution, and robustness, making it a practical choice for real-time energy management applications.

### Performance comparison of load forecasting techniques

**Fig. 5:**
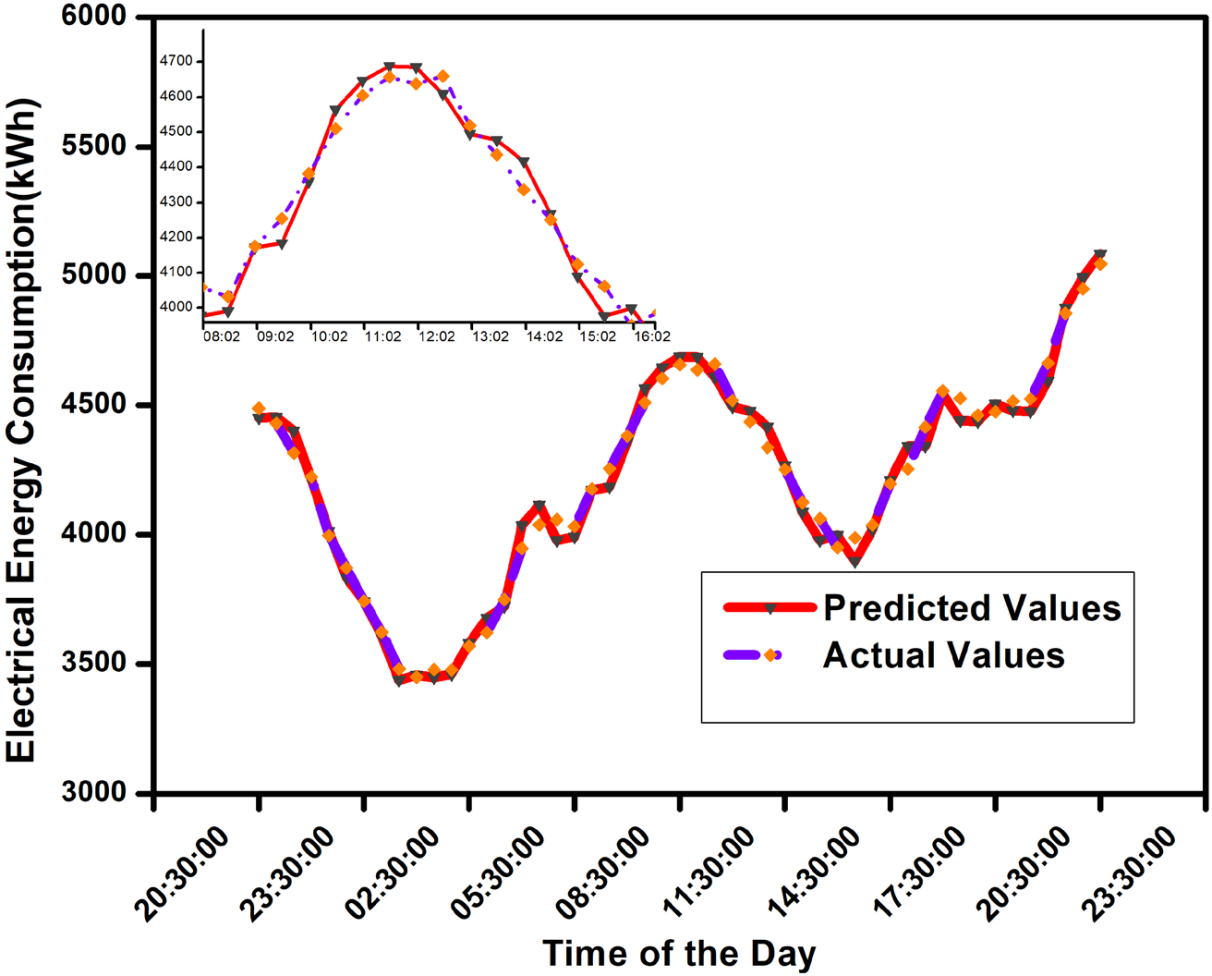
Actual and forecasted load with hour resolution for a day ahead consumption for proposed method.

This section compares the efficacy of various load forecasting techniques, such as the proposed ensemble method with missing data imputation and other baseline techniques. Mean absolute percentage error (MAPE), root mean squared error (RMSE), mean absolute error (MAE), and R2 are the evaluation metrics used to determine the precision and performance of the techniques. Figure 5 shows the actual and forecasted load evaluation for a day ahead time horizon with an hour resolution. The small graph in Fig. 5 shows the zoomed version of the peak power load data of the daytime. The corresponding numerical values of each hour of forecasted and actual load for the ISSDA dataset with the MAPE values are listed in Table 5. The MAPE error value of the proposed model is lower than that of other benchmark models. The MAPE for our method is 0.648% whereas XG-Boost is 4.846%, ARIMA is 3.839%, and 4.876% for LSTM. Lowering the MAPE values results in better accuracy.

**Table 5:** Evaluation of actual and predicted load in terms of MAPE for proposed methods and other state-of-the-art methods XG-Boost, ARIMA, and LSTM.

		XGBoost		ARIMA		LSTM		Proposed	
Hours	Target(KW)	Predicted	MAPE	Predicted	MAPE	Predicted	MAPE	Predicted	MAPE
00.00	4430.629	4343.444	1.968	4235.084	4.413	4276.965	3.468	4455.614	0.564
01.00	4223.342	4002.982	5.218	4114.323	2.581	3963.439	6.154	4212.155	0.265
02.00	3871.777	3685.353	4.815	3807.364	1.664	4037.680	4.285	3834.249	0.969
03.00	3623.767	3333.121	8.021	3611.041	0.351	3902.279	7.686	3611.601	0.336
04.00	3449.702	3311.295	4.012	3273.492	5.108	3236.544	6.179	3457.846	0.236
05.00	3478.004	3249.328	6.575	3382.162	2.756	3230.380	7.120	3460.246	0.511
06.00	3621.963	3494.773	3.5116	3329.651	8.071	3887.300	7.326	3627.829	0.162
07.00	3946.806	3685.038	6.632	3741.931	5.191	4021.165	1.884	3936.517	0.261
08.00	4058.01	3674.883	9.441	3696.962	8.897	4162.304	2.570	3977.005	1.996
09.00	4175.937	4033.329	3.415	4085.891	2.156	3945.143	5.527	4172.433	0.084
10.00	4382.279	4132.179	5.707	4149.224	5.318	4225.964	3.567	4359.732	0.515
11.00	4604.225	4354.885	5.415	4551.218	1.151	4348.110	5.563	4646.425	0.917
12.00	4637.381	4241.467	8.537	4421.044	4.665	4535.903	2.188	4685.434	1.036
13.00	4519.154	4491.836	0.604	4547.497	0.627	4702.769	4.063	4495.285	0.528
14.00	4336.627	4135.329	4.642	4408.397	1.655	4273.974	1.445	4417.578	1.867
15.00	4124.31	3820.124	7.375	3803.447	7.780	4157.889	0.814	4088.936	0.858
16.00	3950.465	3845.243	2.664	4052.333	2.579	4207.946	6.518	3998.481	1.215
17.00	4037.062	3673.524	9.005	3881.926	3.843	4118.591	2.020	4019.234	0.442
18.00	4253.942	4026.001	5.358	4402.410	3.490	4551.048	6.984	4243.254	0.251
19.00	4555.719	4555.159	0.0122	4775.723	4.829	4177.539	8.301	4544.378	0.249
20.00	4462.116	4363.595	2.207	4582.505	2.698	4143.518	7.140	4438.425	0.531
21.00	4516.087	4370.618	3.221	4632.492	2.578	4182.086	7.396	4480.005	0.799
22.00	4662.843	4434.083	4.906	4637.450	0.545	4512.752	3.219	4664.905	0.044
23.00	4949.58	4798.500	3.052	4493.999	9.204	5227.231	5.610	4994.658	0.911
Avg.			4.846		3.839		4.876		0.648

**Fig. 6:**
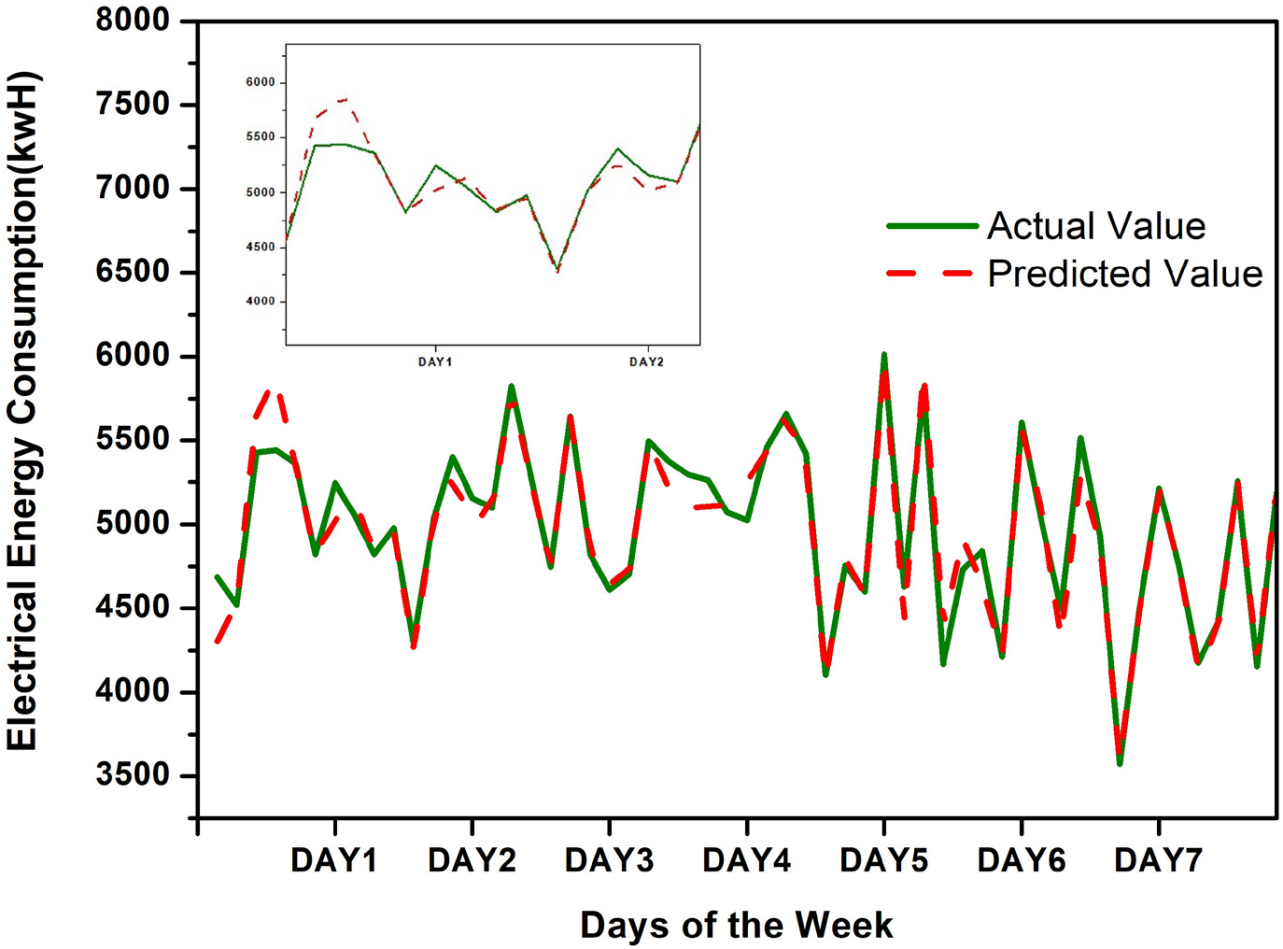
Actual and forecasted load with hour resolution for a day ahead consumption for proposed method.

This model also works well for the seven-day-ahead load prediction. Figure 6 shows the week ahead prediction using the proposed method. It is clear from the figure that predicted values are close to actual values. The small graph in Fig. 6 shows the zoomed version of the power load data for day 1 and day 2.

**Fig. 7:**
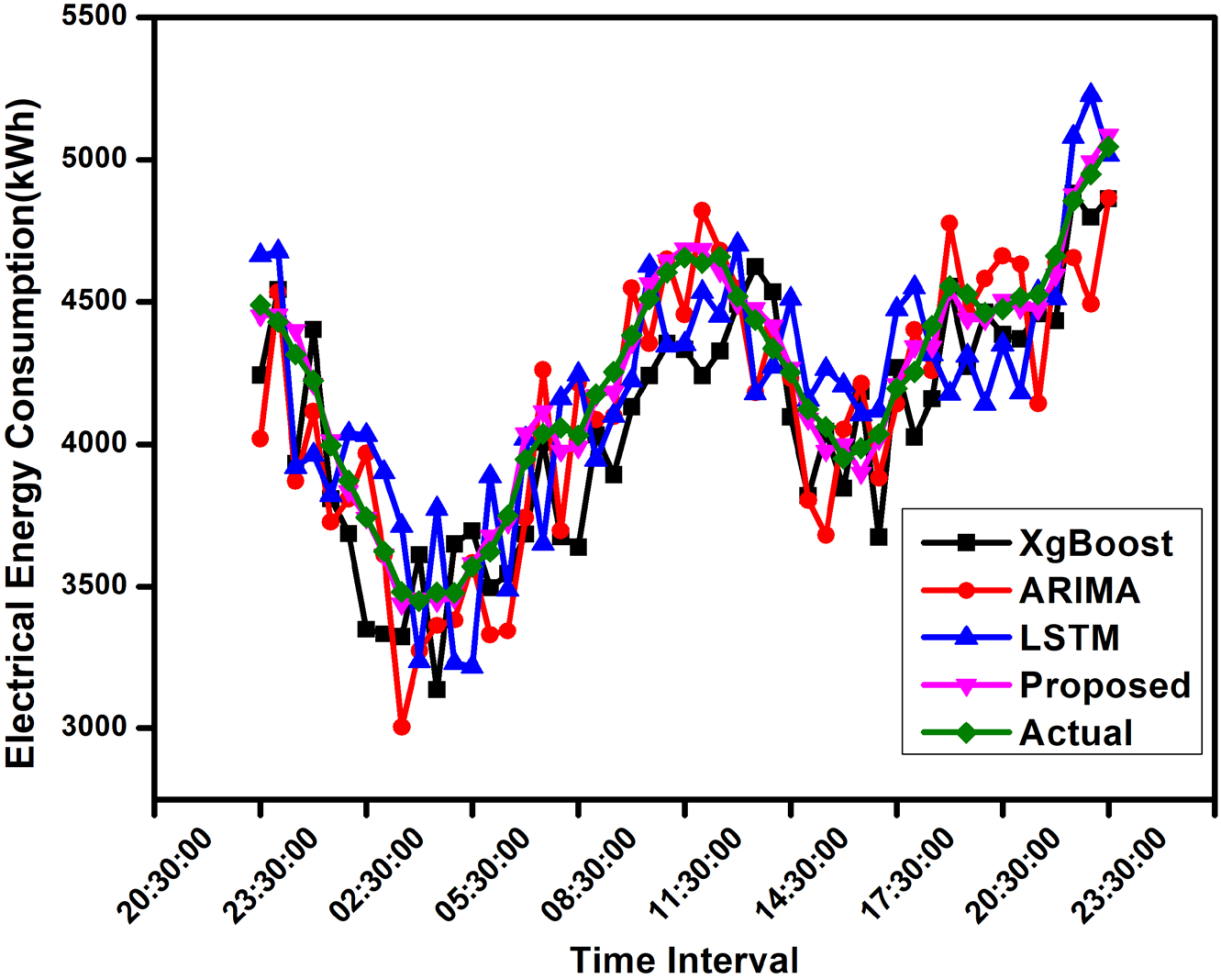
A day-ahead evaluation with the hourly resolution was conducted to assess the forecast accuracy of the proposed Ensemble-based model compared to benchmark models, such as XG-Boost, ARIMA, and LSTM.

Figure 7 compares load forecasting for an intra-day of the proposed EVR method using well-known methods. The figure analyzes the adequacy of the forecasted method compared to other known methods.

**Fig. 8:**
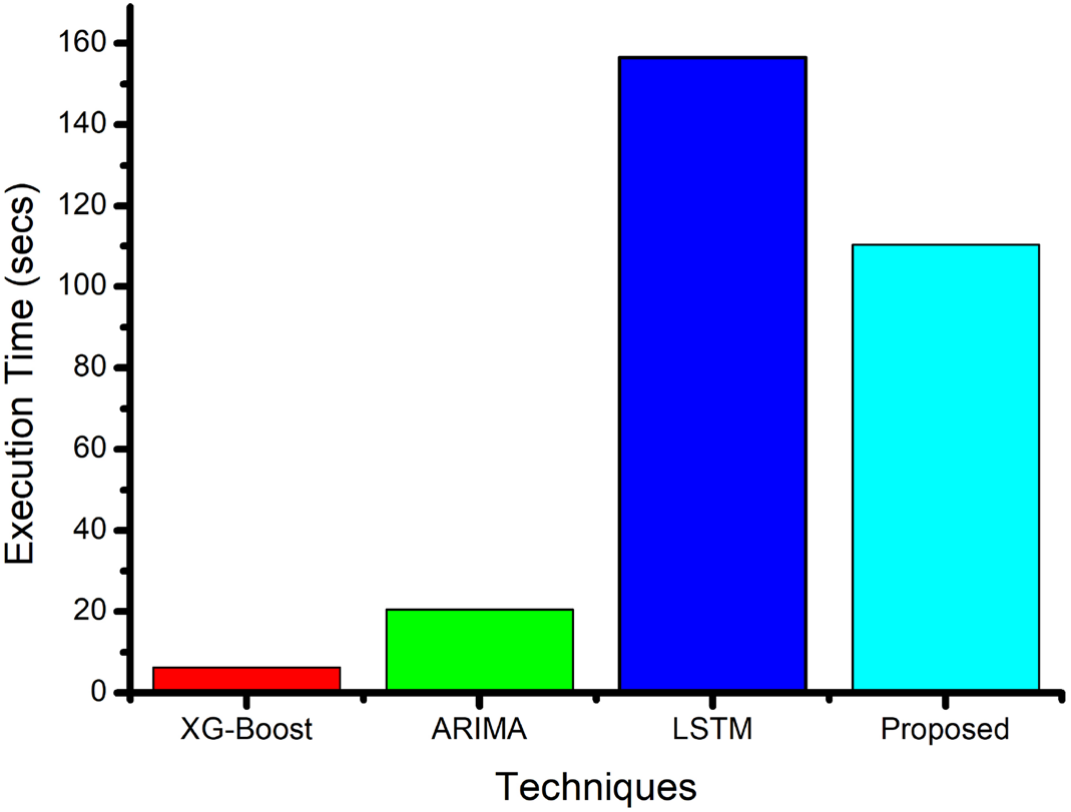
Comparison of Time consumption of XG-Boost, ARIMA, LSTM, and Proposed forecasting methods.

Table 5 shows the predicted consumption value for each time instant for different benchmark techniques with the proposed technique. The predicted value and MAPE score have been evaluated in the table. It is observed that the proposed technique outperforms. Figure 8 shows the execution time required for each method. The bar graph shows that the proposed method takes less time than LSTM but more to XG-Boost and ARIMA models. The proposed ensemble voting regressor is computationally efficient due to the tree-based model and inherent parallelism, and has lower training complexity than deep learning models. A non-iterative inference approach enables low computational cost, making it suitable for time-sensitive applications such as demand response, dynamic pricing, grid balancing, and automated energy scheduling. Unlike deep learning models that often require substantial computational resources, tree-based ensembles are lightweight and efficient, allowing deployment on standard hardware. This makes it feasible to integrate into cost-constrained energy management.

**Table 6:** Perfomance comparison for different load forecasting techniques.

Methods	RMSE	MAE	R2
XGBoost	23.02	5.47	0.36
ARIMA	13.14	8.07	0.06
LSTM	11.36	3.18	0.56
Proposed	3.62	2.46	0.82

Regarding load forecasting, the suggested ensemble technique with missing data imputation has produced encouraging results. In order to increase load forecasting accuracy, spline imputation and ensemble models are used. The ensemble method is compared to several benchmark methods in Table 6 for different evaluation parameters. The performance comparison outcomes in Table 6 show how accurate and dependable the suggested ensemble strategy is. The proposed model has MAPE (0.648%), RMSE (3.62 kW), and MAE (2.46 KW) values that are modestly lower than benchmark methodologies, resulting in significant improvements in the accuracy of grid-level load forecasting overall. This increased precision contributes to better load balancing and optimized scheduling of generation and storage resources. Furthermore, in a smart grid environment, when the decision is made on an hourly basis, even a small increase in prediction accuracy helps utilities to better respond to demand fluctuations, lessen their dependence on reserve power, and improve the cost-efficiency of energy procurement.

## Conclusion

This study proposes a hybrid EVR prediction model that integrates the attributes of three distinct machine learning algorithms to estimate the electricity demand in smart grids for both short-term (one-hour and one-day ahead) and medium-term (one-week ahead) periods with an hour resolution. Furthermore, an imputation technique is developed to address missing values in smart meter datasets, thereby enhancing the accuracy of demand forecasts. The study compared the efficacy of various approaches for missing data imputation and load forecasting within the context of energy management systems. It assesses the fundamental performance metrics such as MAPE, RMSE, and CSS for different imputation techniques. The proposed spline interpolation method for handling missing data thoroughly contrasts with other options like mode, mean, median, LOCF, NOCB, and KNN . The proposed method consistently shows more statistical significance, accuracy, and precision across a range of scenarios with varying percentages of missing data rates from 10 to 30%. The proposed EVR prediction model is tested using the historical Irish Smart Meter Energy Trial dataset, and its performance is validated through comparisons with state-of-the-art forecasting models, such as XGBoost, ARIMA, and LSTM. These baseline prediction models, evaluated using comprehensive metrics like MAPE, RMSE, MAE, and R2 are used to assess the proposed ensemble-based forecasting model. The methodology facilitates making improvements in load forecast accuracy for the coming day and week. The study highlights the accuracy and dependability of the recommended missing data handling strategy by demonstrating its actual use on a particular customer profile. This demonstrates how the method can increase the accuracy of load forecasts and offer more trustworthy information for energy management. The future scope of this work can include the modification of the proposed methodology to extend the forecasting horizon to long-term periods. It would be necessary to modify the current model in order to take into consideration other complexities and factors that affect energy consumption over long periods of time, such as seasonal fluctuations, long-term patterns, and possible changes in consumer behavior. There is possibility to increase predictive accuracy with focus on increasing diversity of base models and exploring more complex ensemble algorithms, such as stacking-based and dynamic weight-based algorithms. Incorporating a probabilistic prediction approach could provide valuable uncertainty estimates for risk-aware decision-making in load prediction tasks. Increasing the number of data samples may have a negative impact on the model’s performance because of specific load curve qualities and characteristics that are susceptible to sudden changes in the weather on the anticipated day. There has to be more research done in this area because these issues could be caused by abrupt variations in the demand for heating or cooling loads.

## Data Availability

Correspondence and requests for materials should be addressed to Sachin Sharma.

## Abbreviations

AMI: Advanced metering infrastructure
ARIMA: Autoregressive integrated moving average
CSS: Cosine similarity score
DT: Decision tree
ET: Extra tree
EV: Electric vehicle
HAN: Home area network
KNN: k-nearest neighbors
LOCF: Last observation carried forward
LSTM: Long short term memory
LF: Load forecasting
LTLF: Long term load forecasting
MAPE: Mean absolute percentage error
ML: Machine learning
MSE: Mean square error
MTLF: Medium term load forecasting
NAN: Neigbourhood area netwrok
NOCB: Next observation carried backward
PARX: Poisson autoregressive with eXogenous covariates
PSO: Particle swarm optimization
R2: R square
RF: Random forest
RMSE: Root mean square error
STLF: Short term load forecasting
SVM: Support vector machine
VSTLF: Very short term load forecasting
WAN: Wide area network
